# Pyrimidine Biosynthesis Is Not an Essential Function for *Trypanosoma brucei* Bloodstream Forms

**DOI:** 10.1371/journal.pone.0058034

**Published:** 2013-03-07

**Authors:** Juma A. M. Ali, Daniel N. A. Tagoe, Jane C. Munday, Anne Donachie, Liam J. Morrison, Harry P. de Koning

**Affiliations:** 1 Institute of Infection, Immunity and Inflammation, College of Medical, Veterinary and Life Sciences, University of Glasgow, Glasgow, United Kingdom; 2 Faculty of Science, Department of Zoology, Al Jabal Al Gharbi University, Gharyan, Libya; 3 Wellcome Trust Centre for Molecular Parasitology, University of Glasgow, Glasgow, United Kingdom; 4 Roslin Institute, University of Edinburgh, Easter Bush, United Kingdom; Louisiana State University, United States of America

## Abstract

**Background:**

African trypanosomes are capable of both pyrimidine biosynthesis and salvage of preformed pyrimidines from the host, but it is unknown whether either process is essential to the parasite.

**Methodology/Principal Findings:**

Pyrimidine requirements for growth were investigated using strictly pyrimidine-free media, with or without single added pyrimidine sources. Growth rates of wild-type bloodstream form *Trypanosoma brucei brucei* were unchanged in pyrimidine-free medium. The essentiality of the *de novo* pyrimidine biosynthesis pathway was studied by knocking out the *PYR6-5* locus that produces a fusion product of orotate phosphoribosyltransferase (OPRT) and Orotidine Monophosphate Decarboxylase (OMPDCase). The pyrimidine auxotroph was dependent on a suitable extracellular pyrimidine source. Pyrimidine starvation was rapidly lethal and non-reversible, causing incomplete DNA content in new cells. The phenotype could be rescued by addition of uracil; supplementation with uridine, 2′deoxyuridine, and cytidine allowed a diminished growth rate and density. *PYR6-5^−^/^−^* trypanosomes were more sensitive to pyrimidine antimetabolites and displayed increased uracil transport rates and uridine phosphorylase activity. Pyrimidine auxotrophs were able to infect mice although the infection developed much more slowly than infection with the parental, prototrophic trypanosome line.

**Conclusions/Significance:**

Pyrimidine salvage was not an essential function for bloodstream *T. b. brucei*. However, trypanosomes lacking *de novo* pyrimidine biosynthesis are completely dependent on an extracellular pyrimidine source, strongly preferring uracil, and display reduced infectivity. As *T. brucei* are able to salvage sufficient pyrimidines from the host environment, the pyrimidine biosynthesis pathway is not a viable drug target, although any interruption of pyrimidine supply was lethal.

## Introduction

Human African Trypanosomiasis (HAT, or sleeping sickness) is caused by infection with the protozoan parasites *Trypanosoma brucei gambiense* and *T. b. rhodesiense* in West Africa and in East and Southern Africa, respectively. In addition the subspecies *T. b. brucei* and the other non-human infective species *T. vivax* and *T. congolense* cause the veterinary condition African Animal Trypanosomiasis (AAT, or nagana) in livestock in much of sub-Saharan Africa. Both diseases continue to have profound health and economic implications in poor and isolated populations of the region. This problem is exacerbated by the inadequacies of the existing drugs, especially their toxicity, and a parenteral route of administration [1], and by high levels of treatment failure that reach about 30% in some areas [2]. The drug of choice for late stage HAT, eflornithine, is currently administered in the form of nifurtimox and eflornithine combination therapy (NECT) and is not suitable for *T. b. rhodesiense* infection, which still has to be treated by suramin or melarsoprol, for Stage I (periphery) or Stage II (central nervous system) disease, respectively; both have severe adverse effects on patients [1], [2]. The use of NECT lowers cost and toxicity but may not halt the spread of eflornithine resistance indefinitely [3], [4]. The quest for new drugs led to the study of nucleotide salvage and biosynthesis in protozoa, and initially focused on inhibitors of purine metabolism, as pathogenic protozoan parasites (but not free-living protists) have lost the *de novo* purine biosynthesis pathways [5], [6]. However, in many protozoa, including kinetoplastid parasites, redundancy of purine transporters [7], [8], [9] and interconversion pathways [10], [11], [12] makes therapy based on purine metabolism inhibitors extremely difficult to achieve.

In contrast, most parasitic protozoa are fully capable of synthesizing the pyrimidine ring *de novo* [10] and yet are also capable of salvaging pyrimidine nucleosides and/or nucleobases [13], [14], [15], [16]. Exceptions are *Plasmodium* spp, which are incapable of pyrimidine salvage [17], and the amitochondriate protozoa *Trichomonas vaginalis*, *Tritrichomonas foetus* and *Giardia* spp, which lack the *de novo* biosynthesis pathway [18], [19]. While possession of both the biosynthesis and salvage routes would appear to make pyrimidine metabolism an unattractive drug target, it has not been established whether either pyrimidine biosynthesis or salvage is essential in African trypanosomes. Moreover, the salvage and biosynthesis pathways actually share most of the pyrimidine metabolizing enzymes, many of which have now been shown to be essential because (in contrast to purine metabolism) there is little or no redundancy in the pathways. For example, dihydrofolate reductase - thymidylate synthase (DHFR-TS) is essential in trypanosomes and its knockout can only be rescued by high levels of thymidine [20], and CTP synthetase is essential as *T. b. brucei* are unable to incorporate extracellular cytosine or cytidine in their nucleic acids [21]. Furthermore, *T. b. brucei* deoxyuridine 5′-triphosphate nucleotidohydrolase (dUTPase) was recently shown to be essential [22] and it is clear that several other enzymes of the same pathways may equally be good drug targets.

However, it is as yet unclear whether either the uptake of extracellular pyrimidines or the *de novo* biosynthesis of the first pyrimidine nucleotide, UMP, is essential in kinetoplastid parasites. We have previously shown that in procyclic *T. b. brucei* pyrimidines are mainly taken up through the TbU1 uracil transporter [23], [16] and recently completed a study of pyrimidine transport activities in bloodstream form *T. b. brucei* showing the presence of only one high affinity uracil transporter, TbU3, and almost no uptake of other pyrimidines at physiological levels [24]. A previous study, by Arakaki *et al* [25] showed that RNAi disruption of one of the biosynthesis enzymes, dihydroorotate dehydrogenase, led to impaired growth which could be compensated for by pyrimidine uptake. The rescue by extracellular uracil, however, was not observed in the presence of the TbU3 inhibitor 5-fluorouracil [25]. In the present study we simulated complete inhibition of pyrimidine salvage by *in vitro* growth in pyrimidine-free medium and inhibition of *de novo* biosynthesis through the construction of a genetic deletion mutant lacking the final step of the pyrimidine biosynthesis pathway, which in trypanosomes is a fusion of the two enzymes Orotidine Monophosphate Decarboxylase (*PYR6*, OMPDCase) and orotate phosphoribosyltransferase (*PYR5*, OPRT) [26], [27]. The *PYR6-5^−/−^* trypanosomes were characterized *in vitro* and *in vivo*. While they were completely non-viable in the absence of pyrimidines *in vitro*, they were able to grow on low levels of pyrimidines, similar as reported for *Leishmania donovani* promastigotes [28]. The activity of the TbU3 transporter, and expression of Uridine Phosphorylase were both significantly increased when *PYR6-5^−/−^* trypanosomes were shifted to pyrimidine-free conditions. However, the observation that these parasites were able to establish an infection in mice showed that pyrimidine biosynthesis is not essential *in vivo*, with pyrimidine salvage from the blood sufficient for *T. brucei* viability and growth.

## Materials and Methods

### Ethics statement

The maintenance and care of experimental animals complied with the appropriate legislation; the UK Animals (Scientific Procedures) Act, 1986, and with the national and University of Glasgow maintenance and care guidelines. All procedures were carried out by trained, registered and licensed animal workers. Care of animals was done by professional staff in the designated University of Glasgow facility under supervision of qualified Veterinarians. Mice infected with trypanosomes were humanely euthanized before becoming seriously ill from the infection. Approval for these experiments was explicitly granted by the UK Home Office, project licence PPL 60/5760 and personal licence PIL60/2328.

### Culture of trypanosomes

Bloodstream forms of *T. b. brucei* strain 427 were routinely cultured in HMI-9 medium [29] obtained from Invitrogen, supplemented with 10% Heat Inactivated Fetal Bovine Serum Gold (FBS; PAA Laboratories) in culture flasks, at 37°C, in a 5% CO_2_ atmosphere. Where indicated, trypanosomes were grown in a pyrimidine-free medium that was identical to the standard HMI-9/FBS, except that it did not contain thymidine (or any other pyrimidines) and that the serum was first thoroughly dialysed (12–14 kDa cut-off) against phosphate-buffered saline pH 7.4); this medium is referred to as HMI-9^-tmd^ whereas the standard medium is simply referred to as HMI-9. The dialysis details and the exact composition of the HMI-9 and HMI-9^-tmd^ media are given in the Supplementary materials.

### Transport of [^3^H]-uracil and [^3^H]-uridine

Transport assays were performed exactly as described [30], [31]. Briefly, trypanosomes were washed into the appropriate assay buffer (AB; 33 mM HEPES, 98 mM NaCl, 4.6 mM KCl, 0.55 mM CaCl_2_, 0.07 mM MgSO_4_, 5.8 mM NaH_2_PO_4_, 0.3 mM MgCl_2_, 23 mM NaHCO_3_, 14 mM glucose, pH 7.3) to a final concentration of 10^8^ cells ml^−1^. 100 µl cell suspension was incubated with either [5,6-^3^H]-uracil (Perkin Elmer, 40.3 Ci/mmol) or [5,6-^3^H]-uridine (American Radiolabeled Chemicals Inc, 30 Ci/mmol) at concentrations indicated in the results section, in the presence or absence of unlabeled substrate or other competitive inhibitors. The incubation was stopped after a predetermined interval using 1 ml of an ice-cold 1-mM solution of unlabeled substrate (uracil or uridine) and immediate centrifugation through oil (13,000×*g*) for 1 min. The resulting cell pellet was transferred to a scintillation tube and radioactivity was determined by liquid scintillation counting. The results were plotted to appropriate equations for linear or non-linear regression using the Prism 5 software package (GraphPad) after correction for non-specific association of radiolabel with the pellet, as described [30].

### Generation of auxotrophic *T. brucei* bloodstream forms

The plasmid pLHTL-PYR6-5 [27] was generously donated by Professor George Cross of Rockefeller University, New York, NY. This construct contains a hygromycin resistance cassette (hygromycin B phosphotransferase) and a negative selection marker, *Herpes simplex* Thymidine Kinase (*HSVTK*) open reading frame between *loxP* domains [32] and is targeted to the *PYR6-5* locus by flanking sequences of 496 bp immediately downstream of the target locus and of 365 bp commencing 134 bp upstream of the ORF.

Bloodstream forms of *T. brucei* s427 were cultivated to a density of ∼1–2×10^7^ cells ml^−1^ and washed into Human T-Cell Solution for transfection with the *LHTL-PYR6-5* cassette (liberated by digestion with *Pvu*II) using an Amaxa Nucleofactor electroporator exactly as described [27], creating a *PYR6-5^+/−^* strain. Transformants were grown and cloned out in standard HMI-9 containing hygromycin (2 µg ml^−1^) and loss of the second *PYR6-5* allele was induced by exposure of the clonal lines to 100 µM 5-fluoroorotic acid (5FOA; Sigma), resulting in a *PYR6-5^−/−^* strain, exactly as described [27], which was cloned by limiting dilution. *PYR6-5* single and double knockout clones were confirmed by PCR. DNA was extracted from WT s427, and from the single and double *PYR6-5* knockout strains using a DNeasy Blood and Tissue Kit (Qiagen). Primers were designed to amplify an 870 bp part of the *PYR6-5* gene. PCR was performed on 200 ng of the isolated DNA using forward (5′ GTTCTCGAGTGCAAGCGGAT) and reverse (5′CACAATGCGGTCAAACTGCA) primers annealing at 56°C and extension at 72°C for 60 s. A Southern blot was also performed to confirm knockouts, using restricted digest of 10 µg DNA and blotting performed as described [33], using DNA probes specific for the *PYR6-5* and hygromycin B phosphotransferase genes. The *PYR6-5* probe was generated using the primers and conditions given above for the PCR confirmation, whilst the hygromycin probe was generated by a PCR using forward (5′ATGAAAAAGCCTGAACTCAC) and reverse (5′ACTCTATTCCTTTGCCCTCG) primers annealing at 55°C and extension at 72°C for 60 s.

### Growth analysis

Growth of WT and *PYR6-5^−/−^* strains was assessed in standard HMI-9 and in HMI-9^-tmd^ supplemented with specific pyrimidines as indicated in the text. Cells were seeded at 1×10^5^ cells ml^−1^ and for this purpose grown in 12-well plates, with each condition set up in 2 wells; incubation was at 37°C and 5% CO_2_. Cells were counted every 12 or 24 h. The experiment was performed independently on three separate occasions.

### Alamar blue drug sensitivity assays

Sensitivities of *PYR6-5^−/−^* and s427-WT cells to 5-fluorouracil, 5-fluoroorotic acid, 5-fluorouridine, 5-fluoro-2′deoxyuridine and 5-fluoro-2′deoxycytidine (all Sigma) were determined using the Alamar Blue assay exactly as described by [34], [35], using a FLUOstar Optima (BMG Labtech, Durham, NC); λ_exc_ was 544 nm and λ_em_ was 620 nm. Seeding density was 10^5^ per well (final volume 200 µl) in doubling dilutions of test compounds and plates were incubated under standard conditions for 48 h, after which the blue, non-fluorescent indicator dye Alamar Blue (resazurin sodium salt; Sigma) was added and the plates were incubated for a further 24 h. Pentamidine was used as positive control throughout, with drug-free incubations as negative control (4 per plate). All drugs were doubly diluted over 23 wells with a starting concentration of test compound of 5 mM for pyrimidines and of 100 µM for pentamidine.

### Flow cytometry

The DNA content of bloodstream trypanosomes was determined exactly as described [36]. Briefly, trypanosome samples were fixed o/n in methanol:PBS (7∶3, v/v), treated with RNase and stained with propidium iodide (both Sigma) and analysed with a FACSCalibur (Benton Dickinson) using the FL2-area detector.

### Quantitative PCR of Uridine Phosphorylase

RNA isolated from WT s427 and *PYR6-5^−/−^* cells was quantified using a Nanodrop (Thermo Scientific); 2 µg of RNA was diluted in RNase-free water to a total volume of 25 µl. Complementary DNA (cDNA) was produced using a ReverseTranscriptase (RT) kit (Primerdesign, UK). cDNA for each sample was diluted 1∶10 and then used for Real Time-PCR. Amplification of cDNA was performed in a 7500 Real Time PCR System (Applied Biosystems). The dissociation curve was used to ensure the amplification of only one product; samples without RT or cDNA were used as controls. The constitutively expressed gene GPI8 [37] was used as endogenous control, with primer sequences 5′- TCTGAACCCGCGCACTTC and 5′-CCACTCACGGACTGCGTTT. For uridine phosphorylase (UP), the ΔΔCT method was used for relative quantification (RQ) using WT cells in HMI-9 as a calibrator or internal control. Data was analyzed using Applied Biosystems 7500 SDS Real-Time PCR systems software. Primers used for the amplification of UP were 5′-TTTGACCCCTCCACCATGA and 5′-GATTCAGCAGGTGAGCCACAA. The entire experiment was performed on three independent occasions, starting from cell culture and RNA isolation.

### Infectivity in mice

Six-weeks-old female ICR (CD-1) Swiss outbred mice (Harlan) were divided into 3 groups of six mice each. Mice were injected intraperitoneally with 10^5^ bloodstream forms of *Trypanosoma brucei brucei* strains .427 WT, *Pyr6-5^+/−^* and *Pyr6-5^−/−^* in 200 µl of HMI-9 medium supplemented with 10% FBS. To quantify parasitaemia, 1 µl of blood was daily harvested by tail venepuncture of each infected mice and appropriately diluted in red blood cell lysis buffer (Sigma). 10 µl of the diluted cells was examined under a light microscope using a haemocytometer and parasitaemia was expressed as number of parasites per ml blood.

## Results

### Generation and confirmation of pyrimidine auxotrophic *T. brucei*

**Figure 1:**
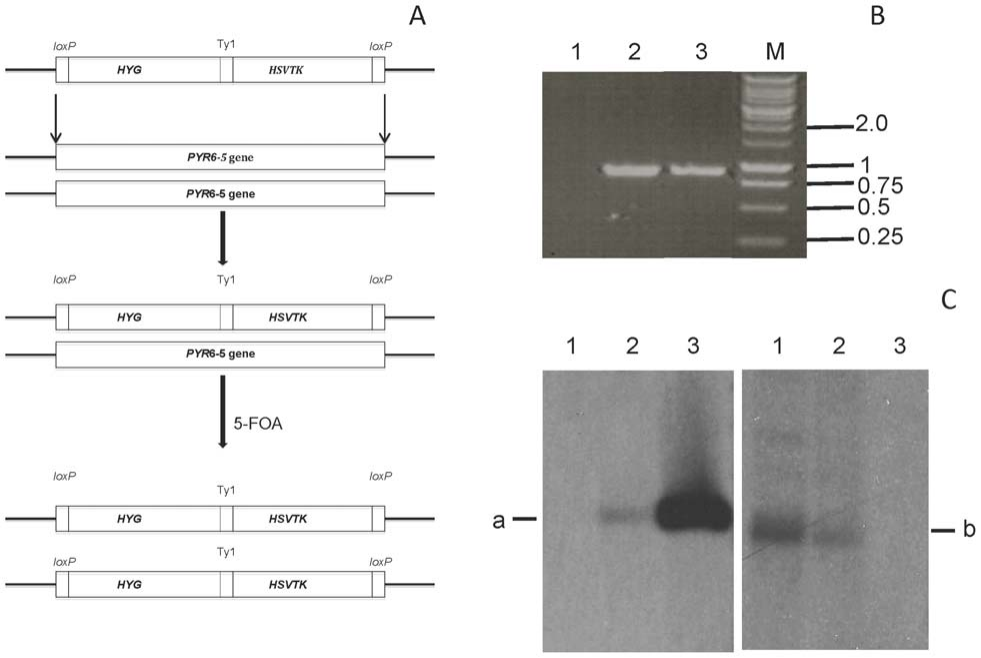
Generation of *Pyr6-5* Knockouts. (A) Schematic representation of the generation of Orotidine Monophosphate Decarboxylase (OMPDCase) knockout. The first step is replacement of one *PYR6-5* allele with a construct containing both positive selection marker hygromycin phosphotransferase (HYG) and negative marker *Herpes simplex* virus thymidine kinase (HSVTK) between 34-bp *loxP* elements. The second step creates the homozygous *PYR6-5* ^−/−^ through drug pressure with 5-fluoroorotic acid (5 FOA), causing loss of heterozygosity. (B) PCR analysis of the *PYR6-5* gene, generating an 870 bp amplicon as described in the Materials and Methods section confirmed the absence of the gene in *Pyr6-5* ^−/−^ (lane 1) and its continued presence in *Pyr6-5* ^+/−^ (lane 2). Lane 3 is the control with WT s427 DNA. (C) Southern blot confirming knockout strategy, using probes for the *PYR6-5* locus and for the HYG-HSVTK cassette. Lane 1, *Pyr6-5* ^−/−^, lane 2, *Pyr6-5* ^+/−^, Lane 3, WT s427. Band ‘a’ is *PYR6-5*, band ‘b’ is *HYG-HSVTK*.

A schematic representation of the generation of a *PYR6-5^−/−^* strain is shown in Figure 1A. Plasmids with the positive selection marker hygromycin phosphotransferase (HYG) and the negative selection marker *Herpes simplex* virus thymidine kinase (HSVTK) (generous donation from George Cross, Rockefeller University, New York) were used. Bloodstream form *T. b. brucei* s427 (1×10^6^ cells ml^−1^) were transformed with the *loxP*-HYG-HSVTK-*loxP* cassette using an Amaxa Nucleofector. The transformants were grown in selective medium containing 4.5 µg ml^−1^ hygromycin (Sigma) and were cloned using limiting dilution, creating a heterozygote *PYR6-5^+/−^* strain. Viable clones with the desired insert were subjected to increasing drug pressure with 5-fluoroorotic acid (5-FOA) leading to loss of the second *PYR6-5* gene (loss of heterozygosity) at 100 µM (Fig. 1B). Loss of heterozygosity (LOH) and the generated homozygous *PYR6-5^−/−^* were further confirmed using Southern blot (Fig. 1C).

### Growth of pyrimidine auxotrophs on different pyrimidine sources

**Figure 2:**
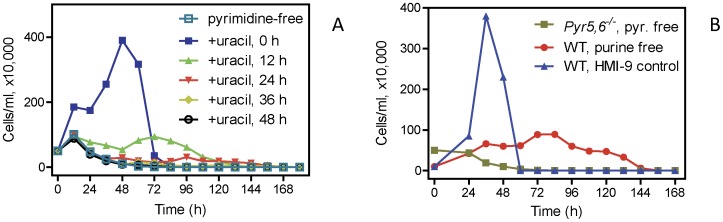
Growth of bloodstream form *T. b. brucei* in media with various purine and pyrimidine content. A. *Pyr5-6^−/−^* trypanosomes were transferred from HMI-9 to HMI-9^-tmd^ (pyrimidine-free, □) medium to which subsequently uracil was added to a final concentration of 100 µM at the indicated time after seeding the culture. Samples were taken every 12 h and cell densities determined using a haemocytometer. In cultures with conditions that allowed fast growth, the trypanosome population declined after 36–48 h due to overgrowth. Cell population in the ‘0 h’ group declined after 60 h due to over-growth and exhaustion of the medium. B. Comparison of purine-free and pyrimidine conditions. WT s427 cells were passaged from mid-log cultures (grown in standard HMI-9 into fresh cultures with the same medium (control,▴) or the same medium without hypoxanthine and supplemented with dialysed serum (purine free, •). Pyrimidine-auxotrophic *T. b. brucei* (*PYR6-5^−/−^*) were transferred from standard HMI-9 into HMI-9^-tmd^ (pyrimidine-free, ▪) medium. Cell population in the ‘WT, HMI-9 control’ group declined sharply after 26–48 h due to over-growth and exhaustion of the media.

Under standard culture conditions there was no clear growth phenotype associated with loss of the *PYR6-5* locus, as growth of the knockout cells in standard HMI-9 was similar to that of WT s427 trypanosomes in HMI-9^-tmd^ supplemented with 10% dialysed FBS (Fig. S1). *PYR6-5^−/−^* cells were grown either in standard HMI-9 or in HMI-9^-tmd^, which does not contain any pyrimidines but does contain 1 mM hypoxanthine as a purine source (Table S1) and is supplemented with FBS that was extensively dialysed to remove small molecules such as nucleosides. As expected, *PYR6-5^−/−^* cells were unable to grow in this semi-defined medium without pyrimidines, and the trypanosome population rapidly declined after 24 h (Fig. 2A,B). In contrast, a shift to purine-free conditions only caused growth arrest after approximately 48 h (Fig. 2B), consistent with previous observations in procyclic *T. brucei* [38]. Evidently, any interruption in pyrimidine supply rapidly makes trypanosomes non-viable and we investigated how quickly the damage becomes irreversible (Fig. 2A), by adding back 100 µM uracil at various times after passage of *PYR6-5^−/−^* to HMI-9^-tmd^. Cells grew to the same density as in standard HMI-9 when uracil was added immediately after passage (0 h control) but adding the uracil after as little as 12 h resulted in irreversible growth arrest and the eventual death of the parasite population. From 24 h, the addition of uracil was almost redundant, with the cell population rapidly declining as in continuously pyrimidine-free conditions (Fig. 2A).

**Figure 3:**
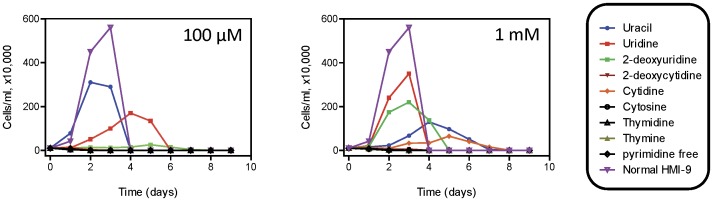
Growth of pyrimidine auxotrophic *T. b. brucei* bloodstream forms on various pyrimidine sources. *PYR6-5* ^−/−^ cell cultures were seeded at a density of 1×10^5^ cells ml^−1^ and cultured at 37°C/5% CO_2_, either in normal HMI-9 or in a simplified pyrimidine version supplemented with dialysed FBS and the indicated pyrimidine source at (A) 100 µM or (B) 1 mM. Samples were taken every 24 h and cell densities determined using a haemocytometer, in duplicate. The experiment shown is representative of several similar experiments with essentially identical results. In cultures with conditions that allowed fast growth, the trypanosome population declined after 36–48 h due to overgrowth.

We next tested the ability of these cells to grow on 100 µM or 1 mM (Fig. 3) of each natural pyrimidine nucleoside or nucleobase. At the lower concentration, uracil supported near-normal growth but at 1 mM appeared to have become somewhat growth inhibitory and growth was less pronounced, possibly because the resulting excessive uracil influx could cause an imbalance between pyrimidine nucleotides and 2′deoxyribonucleotides, or between purine and pyrimidine nucleotides. Uridine also supported growth at 100 µM and even better at 1 mM, whereas 2′-deoxyuridine barely had any effect at all at 100 µM. Of the other pyrimidines, only cytidine had any effect on growth, and only at 1 mM.

### [^3^H]-Uracil uptake in pyrimidine auxotrophic *Trypanosoma brucei*

**Figure 4:**
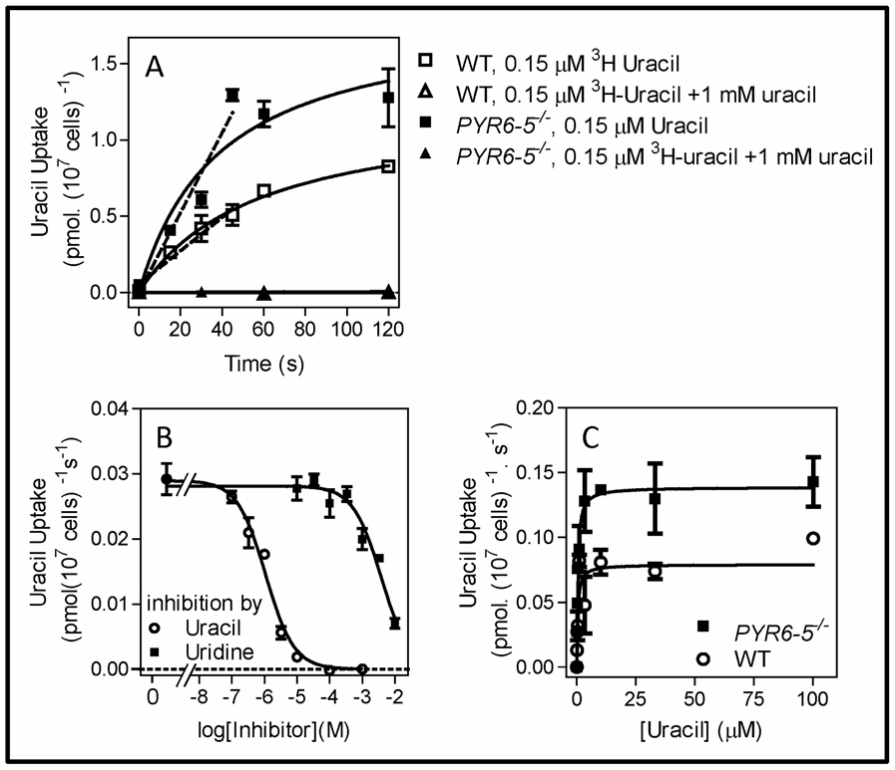
Uracil transport by *T. b. brucei* bloodstream forms. (A) Timecourse of 0.15 µM [^3^H]-uracil uptake by WT s427 and by *PYR6-5* ^−/−^ cells, in the presence or absence of 1 mM unlabelled uracil, as indicated. Dashed lines represent linear regression over the first 50 s, yielding correlation coefficients of 0.97 and 0.92 for the knockout and WT strains, respectively. In the presence of excess unlabelled uracil uptake was not significantly different from zero (F-test) for both strains. The experiment shown is representative of three identical experiments with highly similar outcomes and shows average and SE of triplicate determinations. In the presence of 1 mM uridine, the lines for WT and *PYR6-5* ^−/−^ were superimposed. (B) Uptake of [^3^H]-uracil by *PYR6-5* ^−/−^ trypanosomes was measured over 30 s in the presence or absence of unlabelled uracil (○) or uridine (▪) at the indicated concentrations. The data was plotted to a sigmoid curve with variable slope which in the case of uridine inhibition was set at zero for its minimum. The data are the average and SE of triplicate determinations and the experiment shown is representative of several independent experiments with essentially identical outcomes. (C) Michaelis-Menten saturation plots for uracil uptake of [^3^H]-uracil by WT (○) or *PYR6-5* ^−/−^ (▪) *T. b. brucei* bloodstream forms. The data represents the average and SE of three identical experiments, each performed in triplicate.

We determined uracil uptake rates in *Pyr6-5* ^−/−^ and control s427 WT cells to assess whether uracil uptake capacity in bloodstream form *T. b. brucei* increased in the absence of pyrimidine biosynthesis. WT and *Pyr6-5* ^−/−^ were grown in standard HMI-9 and uptake of 0.15 µM [^3^H]-uracil was measured in a timecourse over 120 s. The initial rate of uracil uptake was consistently higher in the *Pyr6-5* ^−/−^ cells (Fig. 4A). The rates were 0.0109±0.0001 and 0.0241±0.0014 pmol(10^7^ cells)^−1^s^−1^ for WT and *Pyr6-5* ^−/−^ trypanosomes, respectively (n = 3; *P*<0.05, Student's T-test, unpaired). The increased uptake rate could not be attributed to the expression of an additional uracil transporter in the knockout strain not present in the WT cells, as K_m_ values were identical in both strains (0.31±0.01 and 0.34±0.03 µM, respectively (n = 3)) and uracil transport was almost completely insensitive to uridine in both strains (K_i_ values >3 mM, n = 3; Fig. 4B). Indeed, uptake of 2.5 µM [^3^H]-uridine was almost undetectable in both strains (data not shown). However, the V_max_ for uracil transport was significantly increased in *Pyr6-5* ^−/−^ cells (0.14±0.01 versus 0.087±0.007 pmol(10^7^ cells)^−1^s^−1^, respectively; n = 3, *P* = 0.012) (Fig. 4C), consistent with the increased initial rate seen in the time course, and probably reflecting a higher number of the uracil transporter in the plasma membrane rather than the expression of a different or additional transport protein.

### Sensitivity of pyrimidine auxotrophic of trypanosomes to pyrimidine analogues

We tested whether pyrimidine auxotrophs were more sensitive to cytotoxic pyrimidine analogues and found that *Pyr6-5* ^−/−^ cells are approximately one order of magnitude more sensitive to most analogues, including 5-fluorouracil and 5-fluoro-2′deoxyuridine (Table 1). The only exception was 5-fluoroorotic acid, whose action is dependent on OPRT and OMPDCase, and to which the *Pyr6-5* ^−/−^ cells were completely impervious up to 5 mM although WT trypanosomes were sensitive to this compound with an EC_50_ of 13.2±1.2 µM. Interestingly, the *Pyr6-5* ^−/−^ strain was also sensitized to 5-fluorouridine whereas the WT cells were not sensitive to this compound up to the limit tested (5 mM).

**Table 1:** EC_50_ values for some pyrimidine analogues tested on WT s427 and *PYR6-5* ^−/−^ bloodstream forms grown in standard HMI-9, using a standard protocol based on the fluorescent indicator dye Alamar Blue.

	WTs427		*PYR6-5* ^−/−^		RF	*P* value
	AVG ± SE(µM)	n	AVG ± SE(µM)	n		
5-Fluorouracil	35.9±1.5	4	2.3±0.07	4	0.06	<0.001
5-Fluoro-2′deoxyuridine	4.6±0.5	3	0.77±0.10	3	0.16	0.002
5-Fluoro-2′deoxycytidine	43.7±4.4	3	4.6±0.9	3	0.105	<0.001
5-Fluorouridine	>5000	5	472±3	3	<0.09	<0.001
5-Fluoroorotic acid	13.2±1.2	3	>5000	4	>380	<0.001

RF, resistance factor, being the ratio of the EC_50_ values (µM) for knockout over WT strains. P value is based on an unpaired Students t-test.

### Expression of Uridine Phosphorylase in pyrimidine auxotrophs

**Figure 5:**
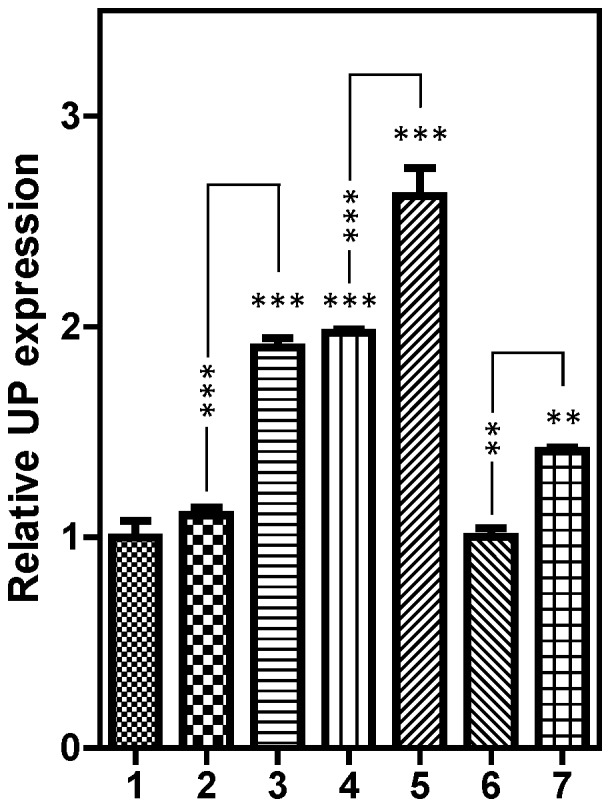
Comparative expression of uridine phosphorylase in wild-type and pyrimidine auxotrophic trypanosomes. Expression of uridine phosphorylase (UP) was assessed by Real Time PCR in WT and *PYR6-5* ^−/−^ strains grown under various conditions. The results are presented normalized to the control (group1) and are the average and SE of 8 replicates. 1. Control: WT grown in HMI-9; 2. WT grown 48 h in HMI-9^-tmd^+100 µM uracil; 3. WT grown 48 h in HMI-9^-tmd^+1 mM uridine; 4. PYR6-5^−/−^ grown 48 h in HMI-9^-tmd^+100 µM uracil; 5. PYR6-5^−/−^ grown 48 h in HMI-9^-tmd^+1 mM uridine; 6. PYR6-5^−/−^long-term adapted to growth on uridine, grown 48 h in HMI-9^-tmd^+100 µM uracil; 7. PYR6-5^−/−^long-term adapted to growth on uridine, grown 48 h in HMI-9^-tmd^+1 mM uridine. Data were analysed with a one-way ANOVA with Tukey's correction. Horizontal asterisks indicate significant differences from control; vertical asterisks indicate significant differences between individual bars as indicated.

We observed that cultures of *Pyr6-5* ^−/−^ cells appeared to be able to adapt to uridine as a sole pyrimidine source (data not shown). In order to utilize uridine for the synthesis of pyrimidine nucleotides they need to generate uracil from it, using uridine phosphorylase (UP) [39]. We thus inferred that upregulation of UP could be a possible adaptation to pyrimidine starvation and performed quantitative PCR to assess relative UP mRNA levels in WT and *Pyr6-5* ^−/−^ cells grown in different media. As shown in Figure 5, UP expression was identical in WT cells grown in standard HMI-9 or in HMI-9^-tmd^ supplemented with 100 µM uracil, but was significantly increased after 48 h growth on HMI-9^-tmd^ supplemented with 1 mM uridine (P<0.001). In *Pyr6-5* ^−/−^ cells cultured long-term in standard HMI-9 but transferred to HMI-9^-tmd^/uracil for 48 h UP expression levels were higher than for s427-WT under the same conditions (P<0.001) and the level was further increased for *Pyr6-5* ^−/−^ cells grown 48 h in HMI-9^-tmd^/uridine (P<0.001). These data appear to indicate that *T. b. brucei* can adjust its UP expression levels to accommodate growth on uridine as its sole pyrimidine source, whether these cells are pyrimidine auxotrophs or prototrophs. We next investigated whether *Pyr6-5* ^−/−^ strains can adapt when long-term cultured on uridine as sole pyrimidine source. We found that these cells do express significantly higher UP levels than s427-WT control cells grown in standard HMI-9 or on HMI-9^-tmd^/uracil (P<0.01), but revert quickly to control levels of expression when shifted to HMI-9^-tmd^/uracil (Fig. 5).

### The effect of pyrimidine starvation on DNA content and integrity of pyrimidine auxotrophic *T. b. brucei*

**Figure 6:**
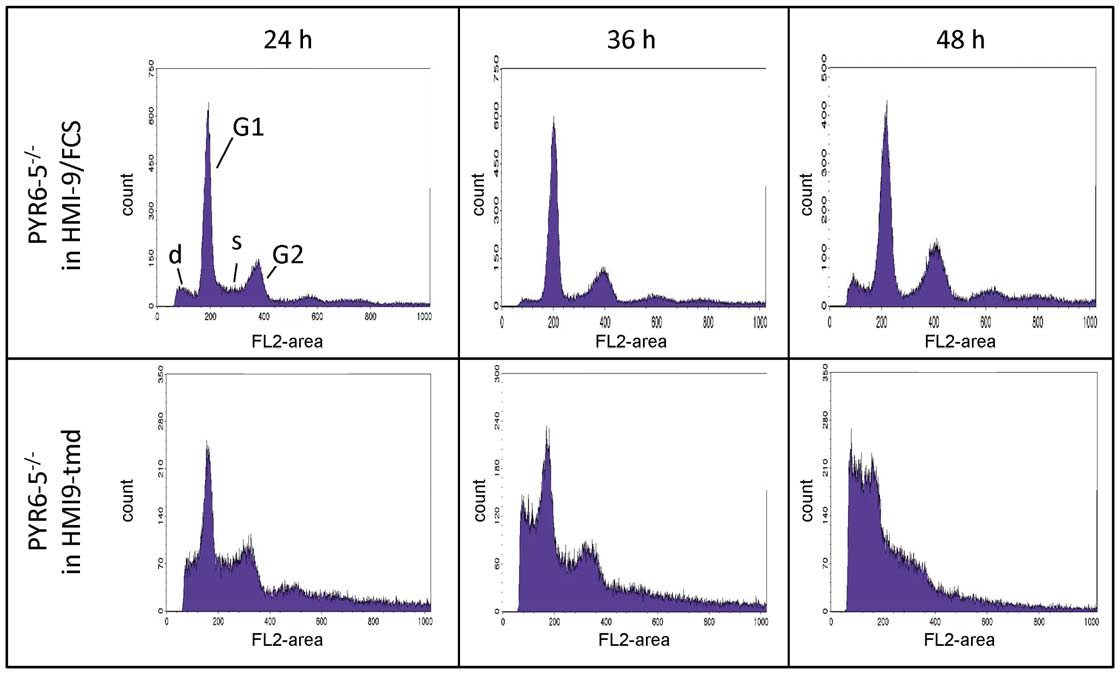
Flow cytometry for DNA content in bloodstream form *PYR6-5* ^−/−^ cells. Pyrimidine auxotrophic trypanosomes were either incubated in standard HMI-9 or, in parallel, in pyrimidine-free HMI-9^-tmd^ for up to 48 h, stained with propidium iodide and prepared for flow cytometric analysis. Whereas control cultures show a classical distribution of cells in G1, S and G2 phase, as well as a small percentage of cells with less than the normal diploid DNA content (debris, d), cells grown in pyrimidine-free medium showed a much higher percentage of cells with partial DNA content, and this increased dramatically between 24 and 48 h.

**Figure 7:**
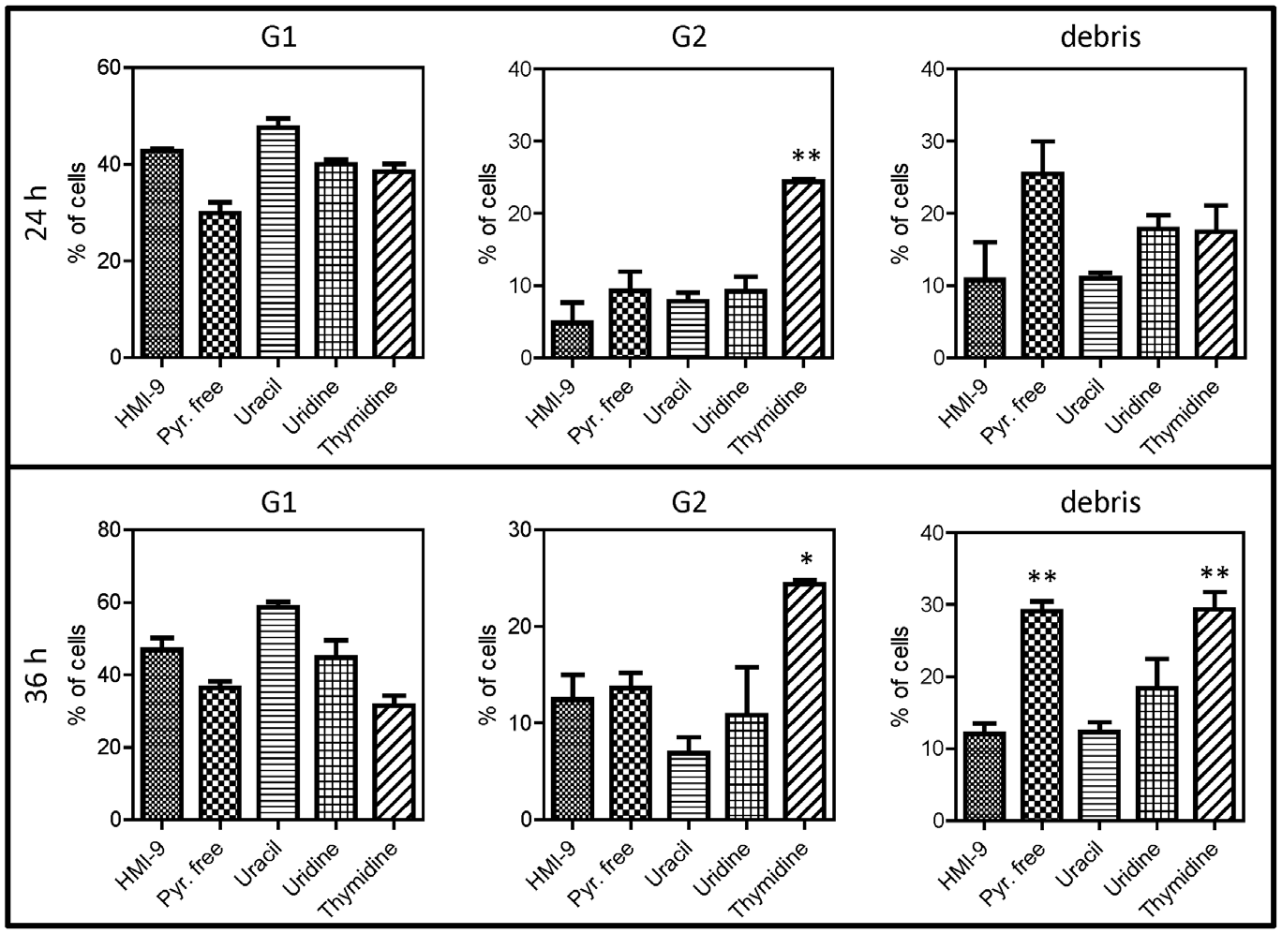
Quantitative analysis of DNA content in pyrimidine-starved trypanosomes. The peak area of G1, G2 and debris of flow cytometric analysis of DNA content (Fig. 6) was determined using the ModFit software package after 24 or 36 of growth under various culturing conditions. Growth was in HMI-9 (control) or in HMI-9^-tmd^ with or without the addition of 100 µM of one pyrimidine as indicated. The data are the average of 3–6 independent determinations and statistical analysis was performed using one-way ANOVA with Tukey's correction (Prism 5, GraphPad). *, P<0.05; **, P<0.01.

Pyrimidine auxotrophs die relatively rapidly in the absence of a salvageable pyrimidine source (uracil>uridine>2′deoxyuridine>cytidine; see figure 3), with death of the population progressing soon after 24 hours. To investigate the cause of the rapid cell death we examined DNA content of the *Pyr6-5* ^−/−^ cells grown in HMI-9^-tmd^ supplemented with 100 µM of various pyrimidines, using flow cytometry with the DNA-binding fluorophore propidium iodide; *Pyr6-5* ^−/−^ cells grown in normal HMI-9 served as control. We found that DNA content in control cells presented a classical distribution of most cells in G1 phase (diploid), a small proportion in S-phase undergoing DNA synthesis and finally a percentage of the population in G2 phase (double set of chromosomes) (Fig. 6). This profile was stable over the 48 hours of the experiment, although the proportion in G2 phase increased somewhat over this period, probably reflecting the mid-log phase of growth of the population at the end of the experiment, compared to early log phase at the start (% in G2 was 4.9±2.8%, 11.6±3.1% and 13.9±1.9% at 24 h, 36 and 48 h, respectively; quantified using the ModFit software package). In sharp contrast, there was a rapid increase in cells displaying an incomplete complement of chromosomes in *Pyr6-5* ^−/−^ cells grown in HMI-9^-tmd^, resulting both in cells with less fluorescence (*i.e.* less DNA) than should be associated with normal G1 phase cells, or cells with a DNA content between G1 and G2 phase (Fig. 6). This clearly indicates that the cells are attempting cell division ‘as normal’ but are unable to complete chromosome synthesis due to lack of pyrimidine nucleotides, leading to aberrant cells with incomplete and fragmented chromosomes that are ultimately non-viable. This phenomenon progressed rapidly and at 48 h few live cells could be detected. The cells that could be counted by the flow cytometer almost all contained incomplete and presumably fragmented DNA. Highly similar flow cytometry results were obtained when supplementing HMI-9^-tmd^ with cytosine, thymine or thymidine, whereas supplementation with uracil or uridine produced profiles highly similar to the control (growth in standard HMI-9); addition of 2′deoxyuridine, cytidine or 2′deoxycytidine resulted in intermediate levels of DNA damage over 48 h (results not shown). In an effort to quantify the emergence of aberrant cells the flow cytometry profiles were analyzed with the ModFit software package which models the peak area. This was not successful for the 48-h time points because of the lack of viable cells and too extensive DNA damage, which did not allow reliable estimates of relevant peaks. However, some results for the 24-h and 36-h time points are shown in figure 7. The use of thymidine as sole pyrimidine source caused a highly significant increase in cells in G2 phase, possibly because of the anticipated imbalance between thymidine nucleotides and deoxycytidine nucleosides, which the cell cannot generate from thymidine. The peak classified as ‘DNA debris’ increased within 24 h of pyrimidine-free conditions and this was highly significant (P<0.01) after 36 h; the debris amount was also significantly increased by culturing on thymidine (Fig. 7). We conclude that any significant interruption of pyrimidine nucleotide availability leads to major defects in DNA synthesis.

### Infectivity of pyrimidine auxotrophic of *T. b. brucei* in mice

**Figure 8:**
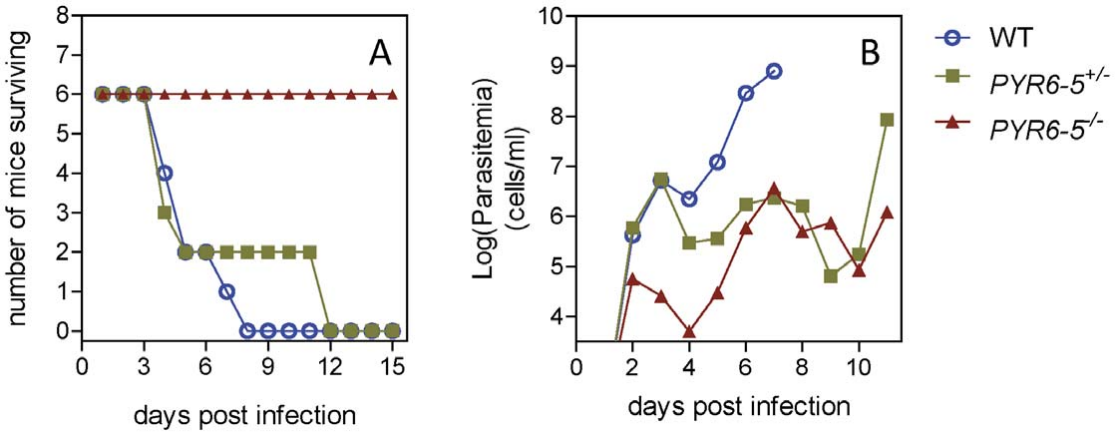
Infectivity of pyrimidine auxotrophic *T. b. brucei*. (A) Survival of mice in groups of 6, each inoculated with 10^5^ bloodstream form trypanosomes of various clonal lines. (B) Parasitaemia of the same mice as depicted for survival in panel A. The average parasitaemia of the surviving mice is shown. Detection was by phase-contrast microscopy and detection limit was 1×10^4^; where the infected was sub-patent, a value of 5000 cells ml^−1^ was inserted in order to arrive at a reasonable average. Both panels: ○, WT s427; ▪, *PYR6-5* ^+/−^; ▴, *PYR6-5* ^−/−^.

The observation (Fig. 3) that pyrimidine auxotrophic *Pyr6-5* ^−/−^ cells grow in standard HMI-9, which contains only thymidine as a pyrimidine source, but cannot grow in thymidine-supplemented medium with dialyzed FBS strongly suggest that (1) *T. b. brucei* cannot use thymidine as its sole pyrimidine source and (2) it is able to salvage sufficient amounts of other pyrimidines from the non-dialyzed serum. It could thus be speculated that pyrimidine auxotrophic trypanosomes should be able to survive *in vivo*. To test this, we infected groups of 6 mice with a high inoculum of 10^5^ trypanosomes of s427-WT, *Pyr6-5* ^+/−^ or *Pyr6-5* ^−/−^ strains and followed survival and parasitaemia for 15 days. Figure 8A shows that WT trypanosomes were the most virulent and killed all mice between 4 and 8 days. The single allele knockout strain *Pyr6-5* ^+/−^ caused the death of four mice by day 5 but two of the animals survived until day 12. In contrast, all the animals inoculated with *Pyr6-5* ^−/−^ survived until day 15, when the experiment was terminated. However, the auxotrophs were able to survive and to multiply in the host, as evidenced by the average parasitaemia, which reached similar levels as for the other strains albeit much more slowly (Fig. 8B). We conclude that *T. brucei* can salvage just enough uracil and/or uridine *in vitro* to maintain an infection.

## Discussion

Kinetoplastid parasites are able to salvage preformed pyrimidine nucleobases and/or nucleosides [6], [7], [15], [40], [41] as well as synthesise them *de novo* from glutamine and aspartate [42]. The two pathways converge at UMP, the end-product of the 6-step biosynthesis pathway as well as the nexus for salvaged cytidine, uridine, 2′dUrd, 2′dCtd and uracil, through the actions of cytidine deaminase, uridine phosphorylase and uracil phosphoribosyltransferase (UPRT). From UMP the cell can then make all pyrimidine ribonucleotides and 2′deoxyribonucleotides that it needs, through non-redundant pathways. Salvaged thymidine can be utilised as thymidine nucleotides but not to produce any other pyrimidine nucleotides ([24]; reviewed in 10 and 19) and both procyclic and bloodstream form trypanosomes take up thymidine very poorly [16], [24]. Therefore it is clear that pyrimidine metabolism in protozoa must be replete with good drug targets. Indeed, *T. b. brucei* DHFR-TS, CTP synthetase and dUTPase have all been shown already to be essential enzymes [20], [21], [22]. These enzymes are all in the pathways downstream from UMP and thus shared by the salvage route and the biosynthesis route.

What is less clear is whether either of the two biochemical pathways to obtain UMP in the first place might be essential and thus a potential drug target. In order to therapeutically target the salvage pathway to UMP it would be necessary to inhibit either the uptake of uracil, uridine, 2′deoxyuridine, cytidine and 2′deoxycytidine, or to inhibit UPRT. With regards to the former option it should be noted that cytidine and 2′deoxycytidine are incorporated very poorly into the *T. b. brucei* nucleotide pool [21], [24] and it would thus only be necessary to inhibit the carriers for uracil, 2′deoxyuridine and uridine, and we recently reported that all three are mediated by the same transporter, TbU3, in bloodstream forms [24]. However, we report here that WT trypanosomes (i.e. pyrimidine prototrophs) grow almost unimpeded in the absence of any pyrimidine source and must conclude that neither pyrimidine transporters nor UPRT are essential functions in bloodstream form *T. b. brucei* - consistent with a recent report that deletion of UPRT in *L. donovani* promastigotes created a pyrimidine prototrophic parasite with normal *in vitro* growth [43]. It can thus be concluded that pyrimidine salvage is not an essential function for trypanosomes.

Arakaki et al [25] previously investigated whether the *de novo* biosynthesis route to UMP was essential to *T. b. brucei* in vitro; they employed RNA-interference (RNAi) to reduce expression of *T. brucei* dihydroorotate dehydrogenase (DHODH). These authors reported that knockdown of this enzyme did not affect growth in standard HMI-9 but greatly reduced growth in pyrimidine-depleted medium using a commercial dialysed serum. Our own observations with a *PYR6-5^−/−^* strain are entirely consistent with Arakaki's report: the growth rate of pyrimidine auxotrophs is at most slightly affected in normal medium with non-dialysed serum. As shown in the Supplementary data, thymidine (∼83 µM) is the only pyrimidine added to that medium but since this nucleoside cannot be converted to uridine and cytidine nucleotides by *T. b. brucei*, it is redundant [24], [25] and clearly the serum provides sufficient pyrimidines for growth, consistent with the average uracil concentration of 0.17±0.05 µM in human plasma [44] and high affinity uptake of pyrimidines, particularly uracil, by *T. brucei* [23], [24]. We thus conclude that pyrimidine biosynthesis is not essential for *in vitro* growth, and the fact that even 10% FBS supplies sufficient pyrimidines, seems to indicate that it may not be essential for *in vivo* growth either.

This was tested by infecting mice with s427-WT, *PYR6-5^+/−^* and *PYR6-5^−/−^* trypanosomes. All three strains were able to maintain an infection and although the homozygous knockout strain was clearly less virulent, it unambiguously establishes that inhibition of the *de novo* pyrimidine biosynthesis is not a viable therapeutic strategy against African trypanosomes. These findings are very similar to those reported for promastigote *L. donovani* [28] but, in contrast to the authors of that report, we contend that the fact that disruption of pyrimidine biosynthesis can be compensated for by physiological levels of pyrimidines demonstrates that this pathway is not essential in kinetoplastids, and not a viable drug target. Indeed, the same authors very recently demonstrated that *L. donovani* that lack carbamoyl phosphate synthetase, and are thus pyrimidine auxotrophic, were able to establish a ‘robust’ infection in mice [43]. It is noteworthy, however, that *Leishmania* species are obligated intracellular parasites and that this manuscript is the first assessment of *in vivo* growth of an extracellular pyrimidine-auxotrophic protozoan. This is relevant as the intracellular and extracellular nucleoside and nucleobase levels are potentially very different, with the intracellular purines and pyrimidines overwhelmingly existing as nucleotides, which cannot be taken up directly by protozoan transporters [6]. In addition, a previous report on pyrimidine-auxotrophic *Toxoplasma gondii*, another obligate intracellular protozoan, showed that these parasites were completely avirulent even in immunocompromised mice [45] - a phenotype attributed to the lack of free pyrimidines within animal cells which also prevents growth of pyrimidine auxotrophic bacteria [46].

Inhibition of the pyrimidine biosynthesis pathway in *T. b. brucei* greatly sensitises the trypanosomes to cytotoxic pyrimidine analogues such as 5-fluorouracil, 5-fluoro-2′deoxyuridine, 5-fluorodeoxycytidine and 5-fluorouridine (Table 1). The enhanced effect of 5-fluorouracil was also noted by Arakaki et al [25], who attributed it to inhibition of uracil uptake. Whilst this analogue is indeed a competitive inhibitor of uracil transport in *T. brucei*, none of these fluorinated pyrimidines would sufficiently inhibit uracil uptake in bloodstream forms at the EC_50_ values given in Table 1, especially not 5F-2′deoxyuridine or 5F-2′deoxycytidine [24]. As an alternative explanation, we propose that these fluorinated pyrimidines enter the trypanosomes as prodrugs and subversive substrates for the pyrimidine salvage enzymes, are converted to nucleotides and incorporated into nucleic acids, as indeed is the case in mammalian cells [47] and as we have recently shown for 5-fluorouracil in *T. b. brucei* [24]. This incorporation is more efficient in the absence of a newly synthesised pool of pyrimidine metabolites that would compete with the halogenated analogues at the level of each enzyme as well as for RNA and/or DNA polymerases.

We thus conclude that an inhibitor of any one of the enzymes of the *de novo* pathway together with either an inhibitor of uracil/uridine uptake, or with a cytotoxic nucleoside analogue, would be a powerful and synergistic combination that would act on trypanosomes through both misincorporation of false nucleotides, and by causing pyrimidine starvation through inhibition of pyrimidine carriers, and we found that trypanosome populations die much more quickly from a lack of pyrimidines than from a lack of purines [38]. It can easily be speculated that kinetoplastid parasites, having evolved without the capacity to synthesise their own purines, must be relatively well-adapted to periods with relatively low purine availability, as demonstrated by the reversibility of purine starvation-induced growth arrest [38]. In contrast, they have not needed to develop a mechanism to cope with a prolonged dearth of pyrimidines, being able to make sufficient amounts themselves, and trypanosomes are therefore unable to recover from even short periods of pyrimidine starvation. We did observe, consistently, increased expression of uridine phosphorylase, and an increase in uracil uptake capacity (both about two-fold), in pyrimidine-starved trypanosomes but this hardly constitutes a major upregulation of the pyrimidine salvage pathway and it is at best uncertain whether this is a regulated, physiological response to low pyrimidine levels. Indeed, the lack of a regulated response to the insufficient level of pyrimidine nucleotides was manifest in the major defects in DNA synthesis after only 24 h, leading to fragmented and incomplete chromosomes.

In summary, we have shown that neither pyrimidine uptake or *de novo* biosynthesis is essential in African trypanosomes but that a drug combination targeting both systems would be a very powerful approach to novel therapeutic approaches against kinetoplastid parasites.

## Supporting Information

Figure S1 **Growth of** ***PYR6-5*** **^−/−^** ***T. b. brucei*** **bloodstream forms in standard HMI-9 and s427-WT in HMI-9-^tmd^ supplemented with 10% dialysed FBS.** Seeding density was 1×10^5^ cells ml^−1^ and cells were manually counted every 24 h. On day 3 cells were passaged to relevant fresh medium, again at 1×10^5^ cells ml^−1^.
(TIF)

Table S1 **Composition of standard HMI-9 medium.**
(DOCX)

Text S1 (DOCX)
