# Supplementary figures and images for: Pyrimidine Biosynthesis Is Not an Essential Function for Trypanosoma brucei Bloodstream Forms
Source: PLoS One. 2013 Mar 7;8(3):e58034. doi: 10.1371/journal.pone.0058034 (PMC3591441; doi:10.1371/journal.pone.0058034)

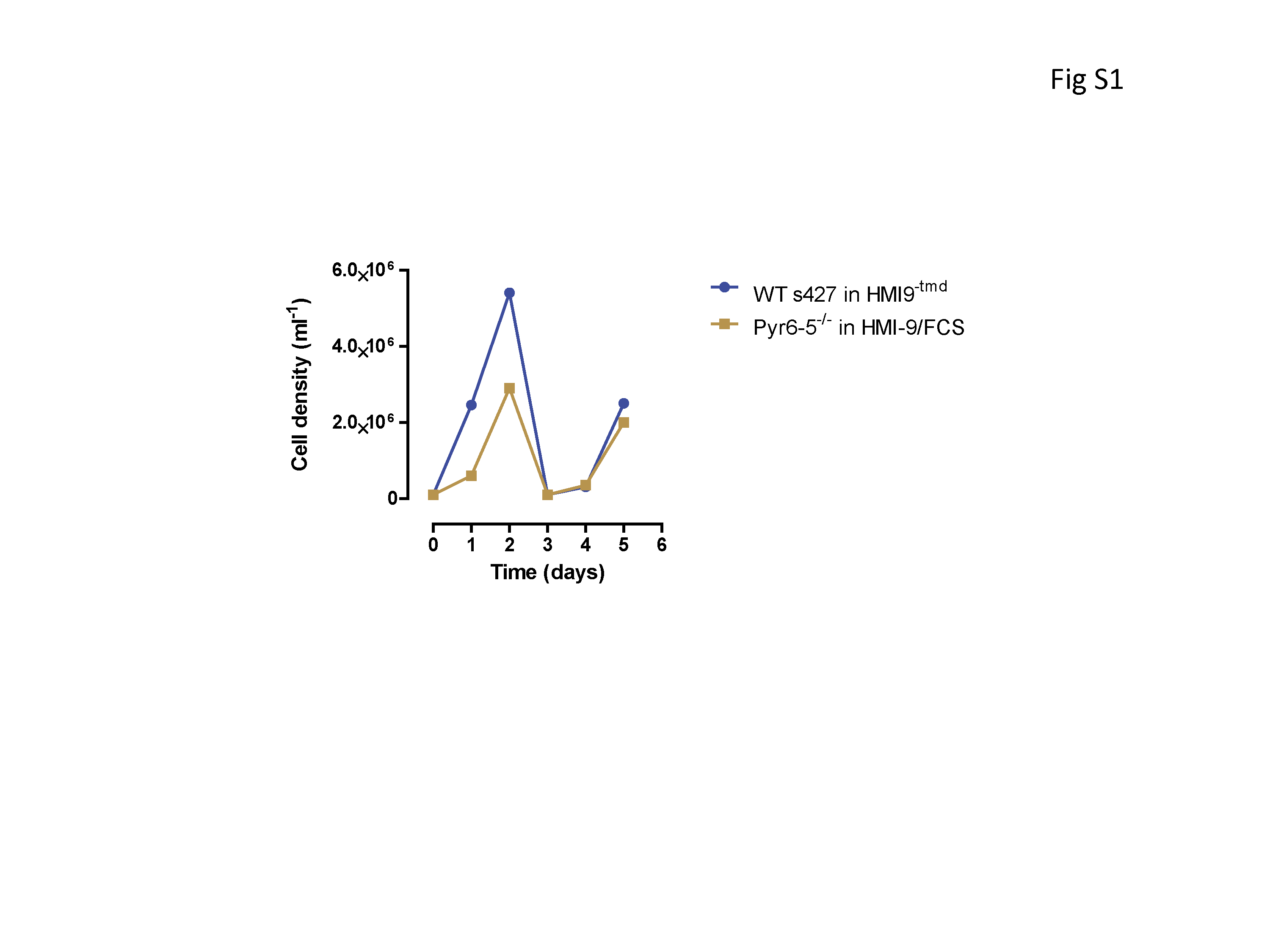

Supplement: Figure S1 — Growth of PYR6-5 −/− T. b. brucei bloodstream forms in standard HMI-9 and s427-WT in HMI-9-tmd supplemented with 10% dialysed FBS. Seeding density was 1×10^5^ cells ml−1 and cells were manually counted every 24 h. On day 3 cells were passaged to relevant fresh medium, again at 1×10^5^ cells ml−1. (TIF) [file pone.0058034.s001.tif]
