# Supplementary material for: Pyrimidine Biosynthesis Is Not an Essential Function for Trypanosoma brucei Bloodstream Forms
Source: PLoS One. 2013 Mar 7;8(3):e58034. doi: 10.1371/journal.pone.0058034 (PMC3591441; doi:10.1371/journal.pone.0058034)
Supplement: Table S1 — Composition of standard HMI-9 medium. (DOCX) [file pone.0058034.s002.docx]

| **Compounds** | **mg/l** |
| --- | --- |
| CaCl_2_ | 165 |
| KCl | 330 |
| KNO_3_ | 0.076 |
| MgSO4 | 97.67 |
| NaCl | 4500 |
| NaHCO_3_ | 3000 |
| NaH_2_PO_4_.H_2_O | 125 |
| Na_2_SeO_3_.5H_2_O | 0.0112 |
| Glucose | 4500 |
| Phenol Red | 15 |
| HEPES | 5958 |
| β-mercaptoethanol^*^ | 14.3 |
| Bathocuproine disulfonic acid, disodium salt | 28.225 |
| Alanine | 25 |
| Arginine HCl | 84 |
| Asparagine | 25 |
| Aspartic acid | 30 |
| Cysteine | 181.74 |
| Cystine | 91.24 |
| Glutamic acid | 75 |
| Glutamine | 584 |
| Glycine | 30 |
| Histidine.HCl.H_2_O | 42 |
| Isoleucine | 105 |
| Leucine | 105 |
| Lysine HCl | 146 |
| Methionine | 30 |
| Phenylalanine | 66 |
| Proline | 40 |
| Serine | 42 |
| Threonine | 95 |
| Tryptophan | 16 |
| Tyrosine | 104.2 |
| Valine | 94 |
| B12 | 0.013 |
| Biotin | 0.013 |
| Calcium d-pantothenate | 4 |
| Choline chloride | 4 |
| Folic Acid | 4 |
| Inositol | 7.2 |
| Niacinamide | 4 |
| Pyridoxal.HCl | 4 |
| Riboflavin | 0.4 |
| Thiamine HCl | 4 |
| Sodium Pyruvate | 220 |
| Hypoxanthine | 136.1 |
| Thymidine | 20.18 |

Table S1. Composition of ‘standard HMI-9 medium.

*, in μL per L
