# Supplementary material for: Pyrimidine Biosynthesis Is Not an Essential Function for Trypanosoma brucei Bloodstream Forms
Source: PLoS One. 2013 Mar 7;8(3):e58034. doi: 10.1371/journal.pone.0058034 (PMC3591441; doi:10.1371/journal.pone.0058034)
Supplement: Text S1 — (DOCX) [file pone.0058034.s003.docx]

*Supplementary notes on media.*

HMI-9 medium was made in our laboratory as itemised in Table S1. The only difference between ‘standard HMI-9’ and ‘HMI-9^-tmd^’ is the omission of added thymidine in the latter. The medium would be made up as thymidine-free and thymidine would be added as required to make ‘standard’ medium. The standard medium was further supplemented with 10% Fetal Bovine Serum, whereas this was first extensively dialysed before addition to HMI-9^-tmd^.

In order to generate purine-free medium we excluded hypoxanthine (136.1 mg/L) from the medium but we added the same amount of thymidine as in the standard medium. Medium was always made up in batched of 5 L.
